# Supplementary material for: A Symbiotic Meal Containing Extruded Sorghum and Probiotic (Bifidobacterium longum) Ameliorated Intestinal Health Markers in Individuals with Chronic Kidney Disease: A Secondary Analysis of a Subsample from a Previous Randomized and Controlled Clinical Trial
Source: Nutrients. 2024 Jun 13;16(12):1852. doi: 10.3390/nu16121852 (PMC11206769; doi:10.3390/nu16121852)
Supplement: Supplementary file 1 [file nutrients-16-01852-s001.zip › nutrients-3025821-supplementary.pdf]

Supplementary Table S1. Nutritional composition of the control and test meals by 100g/portion.

Compounds (g/100g)	Drink Type				
	Extruded Sorghum Meal		Extruded Corn Moisture Meal		
	Mean	SD	Mean	SD	p value
Carbohydrate	33.42	0.29	33.52	0.15	0.61
Lipids	3.36	0.05	3.53	0.04	0.01
Protein	7.90	0.42	8.56	0.33	0.14
Total Dietary Fiber	3.54	0.45	2.91	0.35	0.04
Phenolic Compounds (mg galic acid equivalent/g sample)	44.20	0.84	32.60	0.36	<0.001
Condensed Tannins (catechin equivalent/g sample)	72.78	12.92	0.00	0.00	-
Total Energy Value (kcal/100g)	196.16	0.67	200.25	2.06	0.03

Values expressed in mean ± standard deviation (SD). * Values expressed in dry matter. ** Mean of three replicates. The data were submitted to unpaired t test at 5% probability, in Graphpad prism version 9.0.

Supplementary Table S2. Nutritional composition of sorghum and corn used in test meals.

Variables (g/100g)	Extruded Sorghum		Extruded Corn	
	Mean	SD	Mean	SD
Quantity	6.57	0.28	6.29	0.31
Ash	1.87	0.40	1.66	0.26
Lipids	0.41	0.13	0.81	0.91
Protein	11.26	1.04	12.66	0.81
Total Dietary Fiber	8.84	0.12	7.28	0.870
Soluble Fiber	0.07	0.55	0.87	0.35
Insoluble Fiber	8.78	1.70	6.41	0.90
Resistant Starch	1.03	0.00	0.15	0.02
Carbohydrates	71.04	0.74	71.30	0.39
Phenolic Compounds (mg galic acid equivalent/g sample)	1.10	0.02	0.81	0.01
Condensed Tannins (catechin equivalent/g sample)	0.71	0.08[a]	0.00[b]	0.00
Total Energy Value (kcal·100 g–1)	332.91[b]	1.67	343.13[a]	5.16

Values expressed in mean ± Standart deviation (SD). * Values expressed in dry matter. ** Mean of three replicates. Same letters on the line do not differ by t test for independent samples at 5% probability.

Supplementary Table S3. Stool classification of treatment groups according Bristol Scale.

**Stool Classification**	**Control Group (n=20)**		**Symbiotic Group (n=19)**	
	**Baseline**	**Endpoint**	**Baseline**	**Endpoint**
**1**	2	0	4	0
**2**	7	1	4	1
**3**	4	2	3	4
**4**	6	16	6	10
**5**	0	0	0	2
**6**	1	1	3	2
**7**	0	0	0	0

The stool classification was performed by Bristol Scale.

Supplementary Table S4. Sequencing data at baseline and at the end of 7 weeks of treatment, according to each group.

Treatment		Good's coverage	Raw Sequences	After filtering and cleaning		After normalization	
			Reads	Reads	OTUs	Reads	OTUs
CG	B	0.996 ± 0.001	33048 ± 7202	23497 ± 4614	352 ± 89	13250 ± 29	341 ± 88
	E	0.997 ± 0.001	33897 ± 7469	24501 ± 5103	337 ± 80	13230 ± 43	308 ± 75
SG	B	0.996 ± 0.001	29698 ± 7823	21300 ± 5111	379 ± 121	13246 ± 54	364 ± 115
	E	0.996 ± 0.001	33848 ± 7504	24005 ± 5281	402 ± 99	13227 ± 41	376 ± 104

Values presented in mean ± standard deviation. CG: control group; SG: symbiotic group; B: baseline; E: endpoint.

Embrapa Agroindústria de Alimentos	**RESULTADO DE ANÁLISE**	Página 1 de 1
		Número R015615

DADOS DO CLIENTE

Solicitante:	Eduardo Henrique Miranda Walter
Plano de Ação/Atividade:	02.11.07.020.00.04
Nome do Material:	Leite UFV - último
Número da Requisição:	0065/2015
Data de Entrada:	12/02/2015
Data da Análise:	23/02/2015
Código CRA:	1500759

RESULTADOS OBTIDOS

Nome da Análise	Identificação da Amostra
	Leite 1500759
Enumeração de Bifidobacterium spp (UFC/g)*	$1,9 \times 10^8$
Coliformes a 45º C (UFC/mL)	<3
Coliformes a 35º C (UFC /mL)	>1100
Salmonella sp. (ausência em 25mL)	Ausência
Contagem Padrão em Placas de Aeróbios mesófilas * (UFC/g)	$>2,5 \times 10^6$ estimado

" Valores estimados referem-se a contagens abaixo ou acima dos limites estabelecidos pela metodologia. Os limites estabelecidos são: * entre 25 e 250 UFC/g."

OBSERVAÇÕES

- Referência completa do método utilizado: *Compedium of Methods for the Microbiological Examination of foods* (2001).
- O Resultado da Análise refere-se exclusivamente à amostra ensaiada, sendo o solicitante responsável pela amostragem e coleta do material.
- Este Resultado de Análise só pode ser reproduzido por completo e com autorização deste laboratório.
- Prazo máximo para pedido de contra prova de análise é de 30 (trinta) dias, a partir da data de emissão do Resultado de Análise.

Rio de Janeiro, 09 de março de 2015.

gov.br Documento assinado digitalmente
JANINE PASSOS LIMA
Data: 06/06/2024 17:11:16-0300
Verifique em https://validar.iti.gov.br

Janine Passos Lima da Silva
Responsável Técnica
Laboratório de Microbiologia
"Dispensa assinatura quando consultado eletronicamente"

Empresa Brasileira de Pesquisa Agropecuária – Ministério da Agricultura, Pecuária e Abastecimento
Av. das Américas, 29.501 – Guaratiba – 23020-470 – Rio de Janeiro, RJ – Telefone (0xx21) 3622-9600 – Fax (0xx21) 3622-9713
Homepage: https://www.embrapa.br/agroindustria-de-alimentos - correio eletrônico: ctaa.cra@embrapa.br

SQ 021FA – Revisão 05 – 26/01/2015

Supplementary Figure S1. **Analysis report on the cellular prediction of the probiotic drink containing *Bifidobacterium longum*.** The Embrapa laboratory, where the analysis carried, has an analysis accredited by ISO 17:025

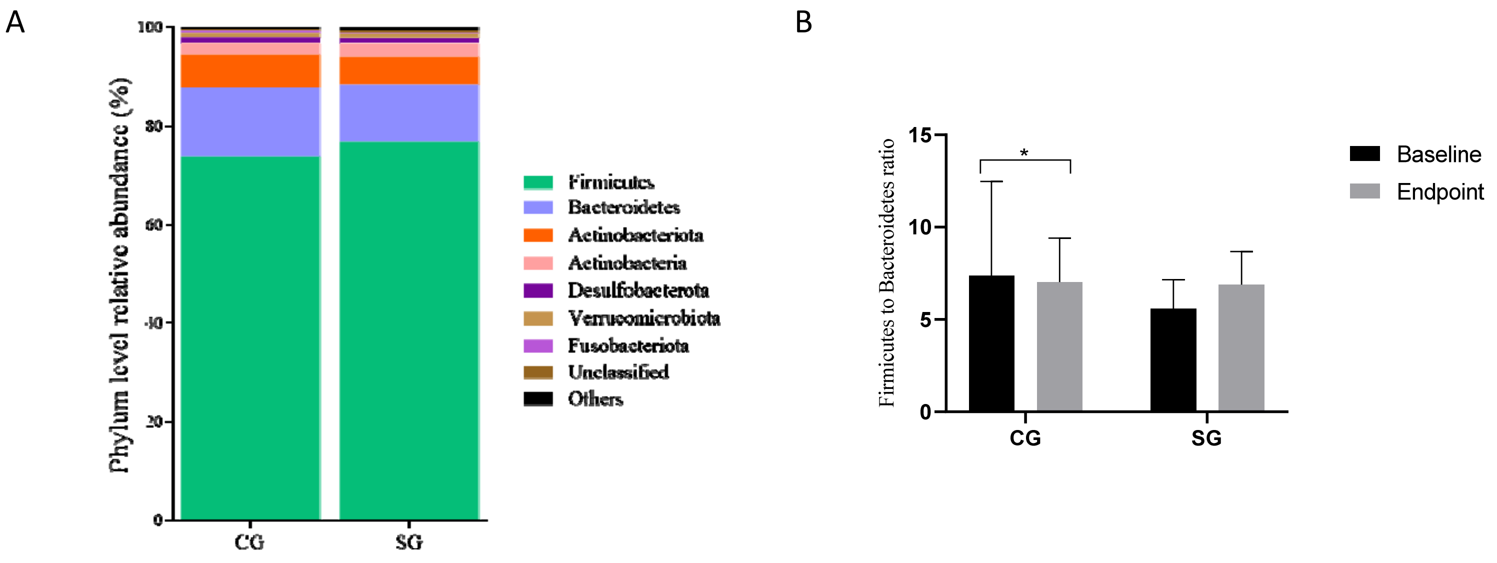

Supplementary Figure S2. Effect of extruded sorghum BRS305 plus *Bifidobacterium longum* on gut microbiota relative abundance at phylum level at the end of treatment. (A) Bacterial composition at phylum level (control group: n=20 ǀ symbiotic group: n=19); (B) Firmicutes to Bacteroidetes ratio at baseline and endpoint. CG: control group; SG: symbiotic group; The data were submitted to paired t-test or unpaired t-test, (α=0.05) in GraphPad version 9.0 and to Dunn's test with FDR and Bonferroni corrections.

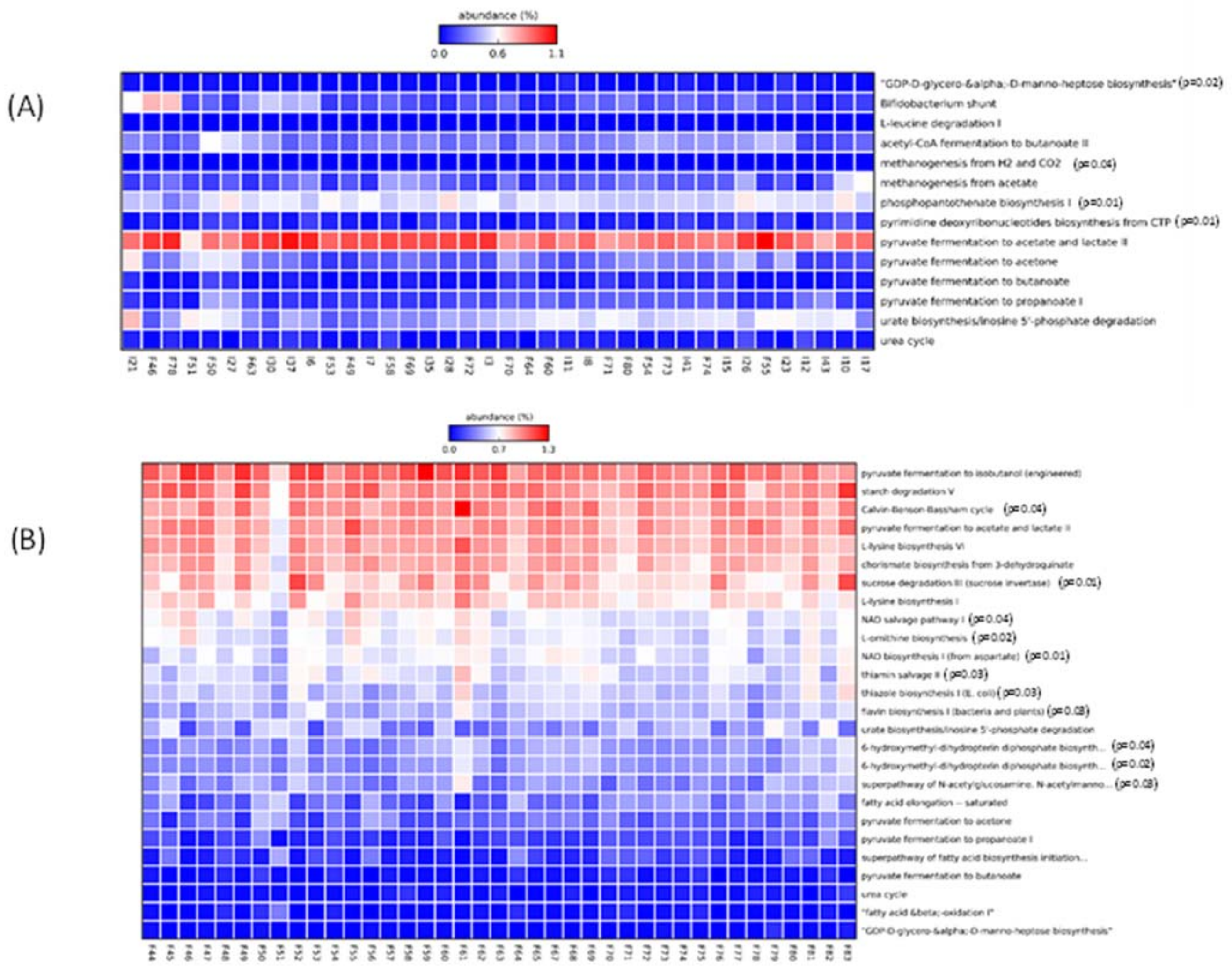

Supplementary Figure S3. Effect of extruded sorghum BRS305 plus *Bifidobacterium longum* in microbial metabolic pathways in feces of CKD patients. (A) Microbial metabolic pathways in feces of CKD patients before and after receiving a symbiotic meal, by the paired t test. (B) Microbial metabolic pathways in the stool of CKD patients that received control drink meal or symbiotic drink meal, by the unpaired t test. Variables presenting differences were pointed out next to the tittle of metabolic pathway. Statistical analyses were performed in the STAMP software system (α=0.05).
